# MiR-125b-5p targets Bak1 to regulate HCT-8 cell apoptosis in response to *Cryptosporidium parvum* infection via mitochondrial pathway

**DOI:** 10.1186/s13071-025-07033-1

**Published:** 2025-10-28

**Authors:** Shanbo Wu, Tianren Shao, Juanfeng Li, Jingjing Xie, Lulu Sun, Yafang Zhang, Lijie Zhao, Xiaoying Li, Junqiang Li, Yayun Wu, Qingda Meng, Longxian Zhang, Rongjun Wang

**Affiliations:** 1 College of Veterinary Medicine, Henan Agricultural University, No. 15 Longzihu University District, Zhengzhou, 450046 China; 2 International Joint Research Laboratory for Zoonotic Diseases of Henan, Zhengzhou, 450046 China; 3 Key Laboratory of Quality and Safety Control of Poultry Products, Ministry of Agriculture and Rural Affairs, Zhengzhou, 450046 China; 4 Ministry of Education Key Laboratory for Animal Pathogens and Biosafety, Zhengzhou, 450046 China

**Keywords:** *Cryptosporidium parvum*, miR-125b-5p, Bak1, Apoptosis, Mitochondrial pathway

## Abstract

**Background:**

*Cryptosporidium parvum* infection can regulate apoptosis of host epithelial cells through both extrinsic and intrinsic apoptotic pathways, potentially involving various microRNAs (miRNAs). This study focuses on the role of miR-125b-5p in modulating apoptosis in HCT-8 cells in response to *C*. *parvum* infection.

**Methods:**

Expression levels of miR-125b-5p and *C*. *parvum* burden were estimated using real-time quantitative polymerase chain reaction (RT-qPCR) and cell apoptosis was detected by flow cytometry. The interaction between miR-125b-5p and Bak1 was studied by luciferase reporter assay, RT-qPCR, and western blotting. The expression of Cytochrome c (Cyt c), Caspase-3, and Caspase-9 were assessed by RT-qPCR, and western blotting. JC-1 assess mitochondrial membrane potential.

**Results:**

MiR-125b-5p expression decreased at 3, 6, 12, and 24 h post-infection (hpi) but increased at 48 and 72 hpi. Upregulation of miR-125b-5p inhibited apoptosis and increased parasite burden in HCT-8 cells following *C*. *parvum* infection, whereas its downregulation promoted apoptosis and reduced the parasite burden. Luciferase reporter assays identified Bak1 as a target gene of miR-125b-5p. Suppression of Bak1 expression via small interfering RNA (siRNA) inhibited apoptosis and increased the *C*. *parvum* burden, while Bak1 overexpression promoted apoptosis and reduced the parasite burden. Co-transfection with miR-125b-5p mimics and pcDNA3.1-Bak1 demonstrated that miR-125b-5p regulates apoptosis and parasite burden by targeting Bak1. Further analysis showed that overexpression of miR-125b-5p significantly decreased the mRNA and protein levels of Cytochrome c (cyt-c), caspase-3, and caspase-9, as well as reduced mitochondrial membrane damage. Inhibition of miR-125b-5p produced opposite effects. Co-transfection with miR-125b-5p mimics and pcDNA3.1-Bak1 revealed that pcDNA3.1-Bak1 counteracted the inhibitory effects of miR-125b-5p on cyt-c, caspase-3, caspase-9, and mitochondrial membrane damage.

**Conclusions:**

The present data indicated that miR-125b-5p targets Bak1 to regulate apoptosis in HCT-8 cells in response to *C*. *parvum* infection via the mitochondrial pathway.

**Graphical Abstract:**

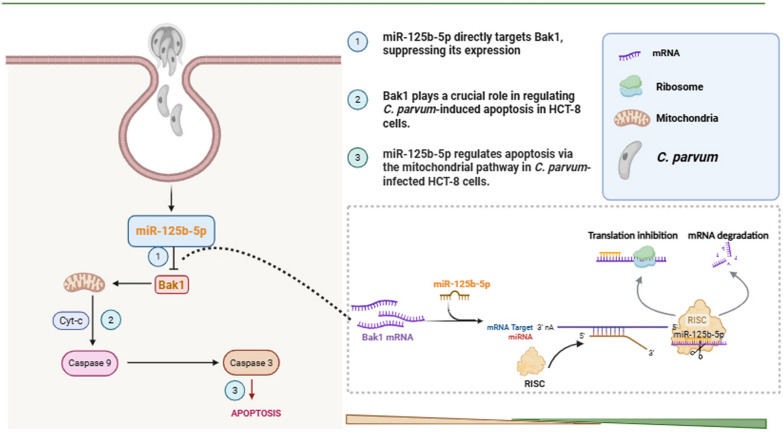

**Supplementary Information:**

The online version contains supplementary material available at 10.1186/s13071-025-07033-1.

## Background

*Cryptosporidium* is an emerging zoonotic protozoan among the most serious global health threats, and ranks as the second leading cause of diarrhea and mortality in children across the world [1]. This pathogen is characterized by its significant genetic diversity, encompassing at least 47 valid species and over 120 identified genotypes. Of these, *C*. *parvum* and *C*. *hominis* are the primary culprits of human infections [2, 3]. Current therapeutic options for cryptosporidiosis are limited. Only nitazoxanide has demonstrated efficacy in immunocompetent individuals, and there is a lack of vaccines available for prevention [4]. Consequently, research into novel drug or vaccine targets is crucial. Further understanding of host-*Cryptosporidium* interactions may yield new strategies for the prevention and control of cryptosporidiosis.

Apoptosis is a genetically regulated and active process of programmed cell death that removes unnecessary or damaged cells, thereby maintaining overall health and stability. Unlike necrosis, apoptosis of intestinal epithelial cells predominantly preserves the integrity of the intestinal barrier [5]. Host cell apoptosis can help resist parasite infections [6]. However, parasites have evolved mechanisms to either induce or circumvent host defenses to survive and complete their life cycle [7, 8]. For instance, in *C*. *parvum*-infected biliary epithelial cells, the NF-κB/IκB signaling pathway is activated to protect cells from death and support parasite survival and reproduction. This regulation forms a part of the parasite’s survival strategy [9]. Nevertheless, the dual-phase modulation mechanism of apoptosis during *C*. *parvum* infection remains unclear, warranting further studies to elucidate host–parasite interactions.

MicroRNAs (miRNAs) are non-coding single-stranded RNA molecules, typically 20 to 23 nucleotides in length, that bind to target mRNAs to either degrade them or inhibit their translation, thereby suppressing target gene expression[10]. In host–pathogen interactions, miRNAs are critical in regulating both innate and adaptive immunity. They influence multiple processes such as activation of intracellular signaling pathways, production of antimicrobial molecules, occurrence of apoptosis and autophagy, and expression of cytokines/chemokines [11–13]. Specifically, host miRNAs are pivotal in regulating the innate immune response to *C*. *parvum* infection [14–18]. For example, miR-26a and miR-30a regulate autophagy via the MAPK signaling pathway in response to *C*. *parvum* infection in vitro [13]. Additionally, *C*. *parvum* infection reduces let-7i expression in host cells through a MyD88/NF-κB-dependent mechanism, enhancing Toll-like receptor 4 (TLR4) expression to facilitate TLR4-mediated defense responses against *C*. *parvum*[19, 20]. Further investigations into miRNA-mediated resistance to *C*. *parvum* infection could suggest new avenues for drug and vaccine development.

Our research group has previously examined miRNA expression profiles in HCT-8 cells during the early stages of *C*. *parvum* infection, identifying several significantly up-regulated or down-regulated miRNAs (e.g., miR-942-5p, miR-181d, miR-125b-5p and et. al). We have reported the roles of miR-942-5p, miR-181d, miR-3976, and miR-199a-3p in apoptosis regulation in response to *C*. *parvum* infection [21–24]. In this study, we focus on the regulatory function of miR-125b-5p in response to *C*. *parvum* infection, aiming to provide new insights into the complex interactions between host miRNAs and *Cryptosporidium*.

## Methods

### Cell culture

HCT-8 human ileocecal adenocarcinoma cells (American Type Culture Collection, Manassas, VA, USA) were cultured in Dulbecco’s Modified Eagle Medium (DMEM) supplemented with 10% fetal bovine serum (FBS), 4 mmol/L l-glutamine, and antibiotics (100 U/mL penicillin and 100 U/mL streptomycin) at 37 °C in a 5% CO_2_ atmosphere. Cells were passaged using 0.25% trypsin (Beyotime, Shanghai, China) upon reaching approximately 80% confluence. Only cells from passages 3 to 6 were utilized for experiments.

### Parasite culture

Newborn calves were sourced from Ruiya Pasture Co., Ltd. (Zhengzhou, China). Calves were infected with *Cryptosporidium parvum* IIaA19G1 subtype oocysts, which were purified and stored in 2.5% K_2_Cr_2_O_7_ at 4 °C. Oocyst excystation was performed using a solution containing 0.25% trypsin and 0.75% sodium taurocholate for 1 h, with mixing every 5 min, followed by a 30-min incubation at room temperature. The oocysts were washed thrice with phosphate-buffered saline (PBS) and the resulting sporozoites were suspended in the culture medium, and sporozoites were then added to a monolayer of cells in either a 6- or 12-well plate in a sporozoite:cell ratio of 4:1.

### RT-qPCR

HCT-8 cells were washed three times with PBS, and total RNA was extracted using TRIzol reagent (Invitrogen, Waltham, MA, USA) per the manufacturer’s instructions. Extracted RNA was treated with recombinant DNase I (Takara, Kyoto, Japan). Reverse transcription into cDNA and miRNA was performed using the ReverTra Ace qPCR RT Master Mix with gDNA remover (Toyobo, Japan) and the Mir-X miRNA First-Strand Synthesis Kit (Takara Biomedical Technology, Dalian, China), respectively. GAPDH and U6 served as reference genes for cDNA and miRNA quantification. Gene expression was quantified using SYBR^®^ Green Real-time PCR Master Mix (Toyobo). RT-qPCR included a melting curve analysis and followed the cycle program: initial activation at 95 °C for 30 s, followed by 40 cycles of 95 °C for 5 s, 55°C for 10 s, and 72 °C for 15 s. Gene expression fold changes were calculated using the 2^−ΔΔC*t*^ method, with control group comparisons shown on the Y-axis. Primer sequences are detailed in Additional file 2: Table S1.

### Cell transfection

MiR-125b-5p mimics, inhibitors, and a negative control (NC) were obtained from GenePharma (Shanghai, China). The Bak1 overexpression vector was constructed in the pcDNA3.1 eukaryotic expression plasmid (pcDNA3.1-Bak1), with the plasmid map presented in Additional file 2: Fig. S1. SiRNAs targeting Bak1 mRNA (si-Bak1) and scrambled RNA (si-NC) were designed by Sangon Biotech (Shanghai, China). Transfection was performed with Lipofectamine 2000 reagent (Invitrogen, Carlsbad, CA, USA) following the manufacturer’s protocol. MiR-125b-5p mimics and pcDNA3.1-Bak1 were co-transfected in equal proportions. The sequences for siRNA, miRNA mimics, and inhibitors are listed in Additional file 2: Table S2. Transfection efficacy of the miR-125b-5p mimic, miR-125b-5p inhibitor, si-Bak1, or si-NC using Lipofectamine 2000 on cell activity is illustrated in Additional file 2: Fig. S2.

### Cell apoptosis flow cytometry

HCT-8 cells infected with *Cryptosporidium parvum* for 24 h post-infection (hpi) were harvested by washing with PBS and treating them with trypsin (without EDTA) for 5 min, followed by centrifugation at 800×*g* for 5 min at room temperature. The supernatant was discarded, and the cell pellet was washed twice with PBS. Cells were then resuspended in 500 µL of pre-cooled 1× binding buffer at a density of 10^5^–10^6^/mL using the Annexin V-FITC/PI Apoptosis Detection Kit (A211) (Vazyme Biotech Co., Ltd, China). Following this, 5 µL of Annexin V-FITC and 5 µL of PI Staining Solution were added. The suspension was incubated for 15 min at room temperature in the dark. Apoptosis was analyzed using a flow cytometer (Beckman Coulter, Brea, CA, USA), with the apoptosis rate calculated as the sum of the early (O1-LR) and late apoptotic cells (O1-UR). All experiments were conducted in triplicate.

### Dual-luciferase reporter assay

The 3′-UTR sequences for wild-type Bak1 (Bak1-WT) and mutant Bak1 (Bak1-MUT) were cloned into the pmirGLO vector (Promega, Madison, WI, USA). The sequences are detailed in Additional file 2: Table S3 and illustrated in Additional file 2: Fig. S3. These constructs were co-transfected into HCT-8 cells with miR-125b-5p mimics or a negative control (NC) using Lipofectamine 2000 (Invitrogen, Gaithersburg, MD, USA). After 48 h, cell lysates were analyzed using the Dual-GLO^®^ Luciferase Assay System (Promega). Relative luciferase activity was determined by normalizing firefly luciferase to Renilla luciferase activity.

### Western blotting

Total protein was extracted from 10^7^ cells using a total protein extraction kit (Solarbio Life Sciences, Beijing, China), and concentrations were determined using the Pierce BCA Assay Kit (Thermo Fisher Scientific, Waltham, MA, USA). Protein samples (20 µg) were separated via 10% SDS-PAGE and transferred to polyvinylidene fluoride membranes. Membranes were blocked with 1% bovine serum albumin and incubated overnight at 4 °C with primary antibodies: Bak1, Caspase-3, Caspase-9 (1:1000, Cell Signaling Technology, Boston, MA, USA), Cytochrome c (1:1000, Abcam, Cambridge, UK), and GAPDH (1:2000, Immunoway, Tennyson, TX, USA). Secondary antibodies conjugated with horseradish peroxidase (1:5000, Immunoway) were then applied for 2 h at room temperature. Protein bands were visualized using enhanced chemiluminescence (ECL) and quantified with ImageJ software.

### JC-1 staining assay

To assess mitochondrial membrane potential changes, a JC-1 Mitochondrial Membrane Potential Assay Kit (Abbkine, Inc, China) was used. HCT-8 cells (2.5 × 10^6^) were seeded in 6-well plates, transfected with miR-125b-5p or Bak1 mimics/inhibitors for 24 h, and then treated with *C*. *parvum* for 12 h. Cells were stained with JC-1 solution (1×) for 15 min and observed under a laser confocal microscope with 585/590 and 510/527 nm filters.

### Statistical analysis

Statistical analyses were performed using GraphPad Prism 8.0.2 (GraphPad Software, San Diego, CA, USA). Differences between two groups were evaluated using an unpaired *t*-test with Bonferroni correction, while non-parametric one-way ANOVA was employed for multiple group comparisons. Results are reported as mean ± standard deviation (SD), with statistical significance set at *P* < 0.05. All experiments included at least three biological replicates.

## Results

### *C*. *parvum* infection modulates miR-125b-5p expression in HCT-8 cells

**Fig. 1:**
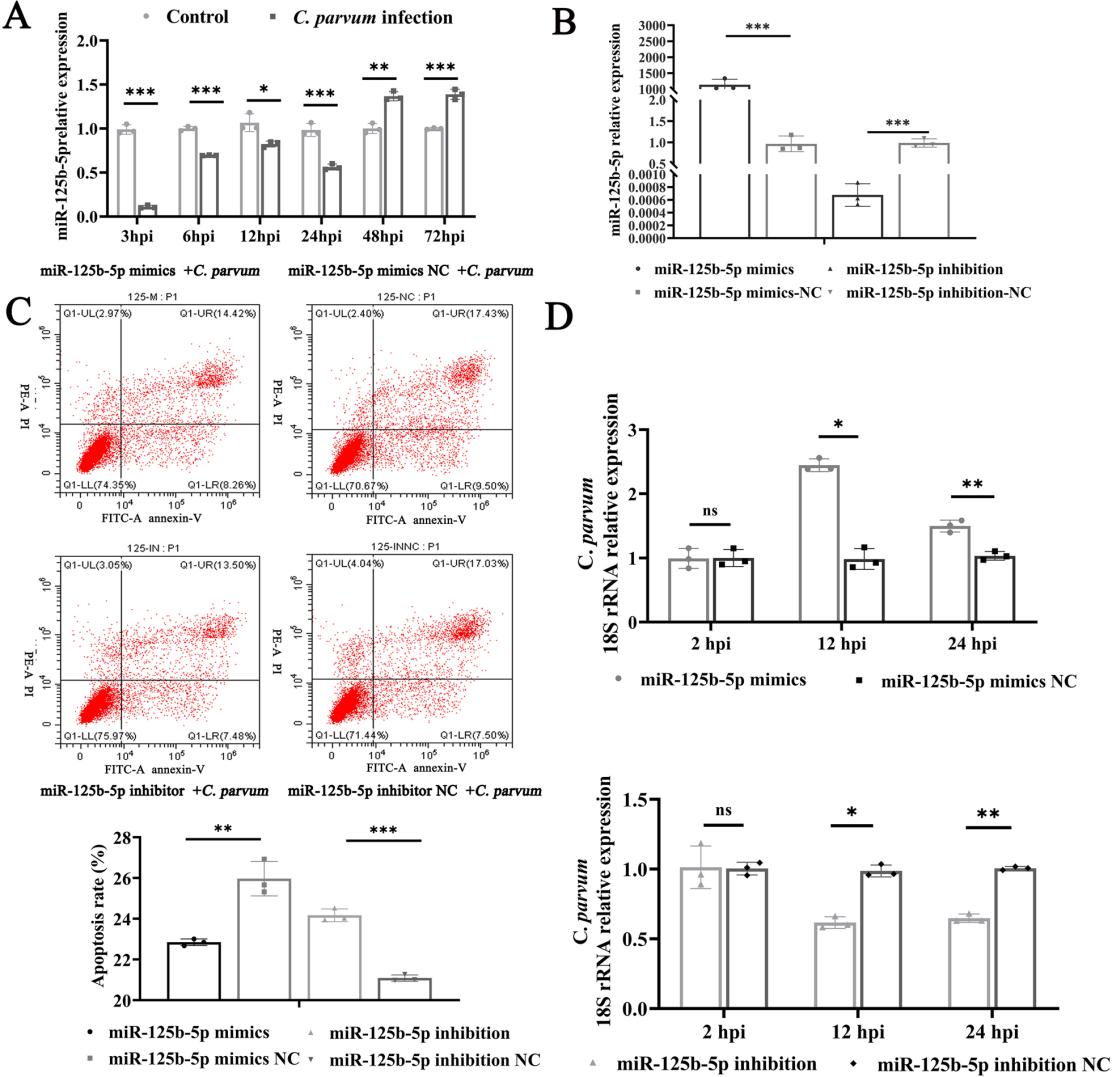
MiR-125b-5p regulates apoptosis and parasite burden in HCT-8 cells infected with *C*. *parvum*. **A** Expression levels of miR-125b-5p in HCT-8 cells at different times after *C*. *parvum* sporozoites infection, determined with RT-qPCR. **B** Expression efficiency of miR-125b-5p in HCT-8 cells transfected with miR-125b-5p mimic or miR-125b-5p inhibitor for 24 h, determined with RT-qPCR. **C** Effect of miR-125b-5p on apoptosis of cells infected with *C*. *parvum*. Cells were transfected with miR-125b-5p mimic or miR-125b-5p inhibitor for 24 h and then exposed to equal numbers of *C*. *parvum* sporozoites for 12 h. Cell apoptosis was detected with flow cytometry. The horizontal axis of each apoptosis diagram is Annexin V and the vertical axis is PI; and the apoptosis rate is the sum of O1-LR and O1-UR. **D** Cells were transfected with miR-125b-5p mimic or miR-125b-5p inhibitor for 24 h and then exposed to equal numbers of *C*. *parvum* sporozoites for 2 h to determine the initial attachment and cellular invasion of *C*. *parvum* with RT-qPCR. The medium was replaced with fresh medium at 2 hpi infection with *C*. *parvum* and the infected cells cultured for a further 12 or 24 h to evaluate the parasite burden after invasion. All data presented are the means ± SD of three independent experiments. Different groups were compared with a *t* test (*P < 0.05, **P < 0.01, ***P < 0.001)

The expression of miR-125b-5p in HCT-8 cells at 3, 6, 12, 24, 48, and 72 h post infection (hpi) following *C*. *parvum* infection was measured by using RT-qPCR. The analysis revealed a significant downregulation of miR-125b-5p at 3, 6, 12, and 24 hpi, followed by a notable upregulation at 48 and 72 hpi (Fig. 1a).

### miR-125b-5p regulates apoptosis and parasite burden in *C*. *parvum*-infected HCT-8 cells

Previous studies have highlighted the pivotal role of miRNAs in modulating host cell apoptosis. To elucidate the role of miR-125b-5p in apoptosis regulation within HCT-8 cells, we transfected cells with either an miR-125b-5p mimic to upregulate or an miR-125b-5p inhibitor to downregulate its expression (Fig. 1b). Apoptosis levels were subsequently evaluated at 12 hpi with *C*. *parvum*. Flow cytometry results showed a significant increase in apoptosis in HCT-8 cells transfected with the miR-125b-5p inhibitor compared with those transfected with the negative control, while cells treated with the miR-125b-5p mimic exhibited markedly reduced apoptosis. This confirms that miR-125b-5p inhibits apoptosis in HCT-8 cells induced by *C*. *parvum* infection (Fig. 1c). Given that apoptosis regulation might correlate with parasite survival strategies, we investigated how miR-125b-5p expression influences the *C*. *parvum* infection burden using RT-qPCR. At 2 hpi, no changes in parasite load were observed, suggesting that miR-125b-5p expression does not affect *C*. *parvum* adhesion or invasion. However, at 12 and 24 hpi, cells with downregulated miR-125b-5p (inhibitor group) exhibited a significant reduction in parasite load, while cells with upregulated miR-125b-5p (mimic group) showed a significant increase in parasite load (Fig. 1d). These findings suggest that miR-125b-5p enhances the *C*. *parvum* burden in HCT-8 cells.

### Bak1 as a target gene of miR-125b-5p

**Fig. 2:**
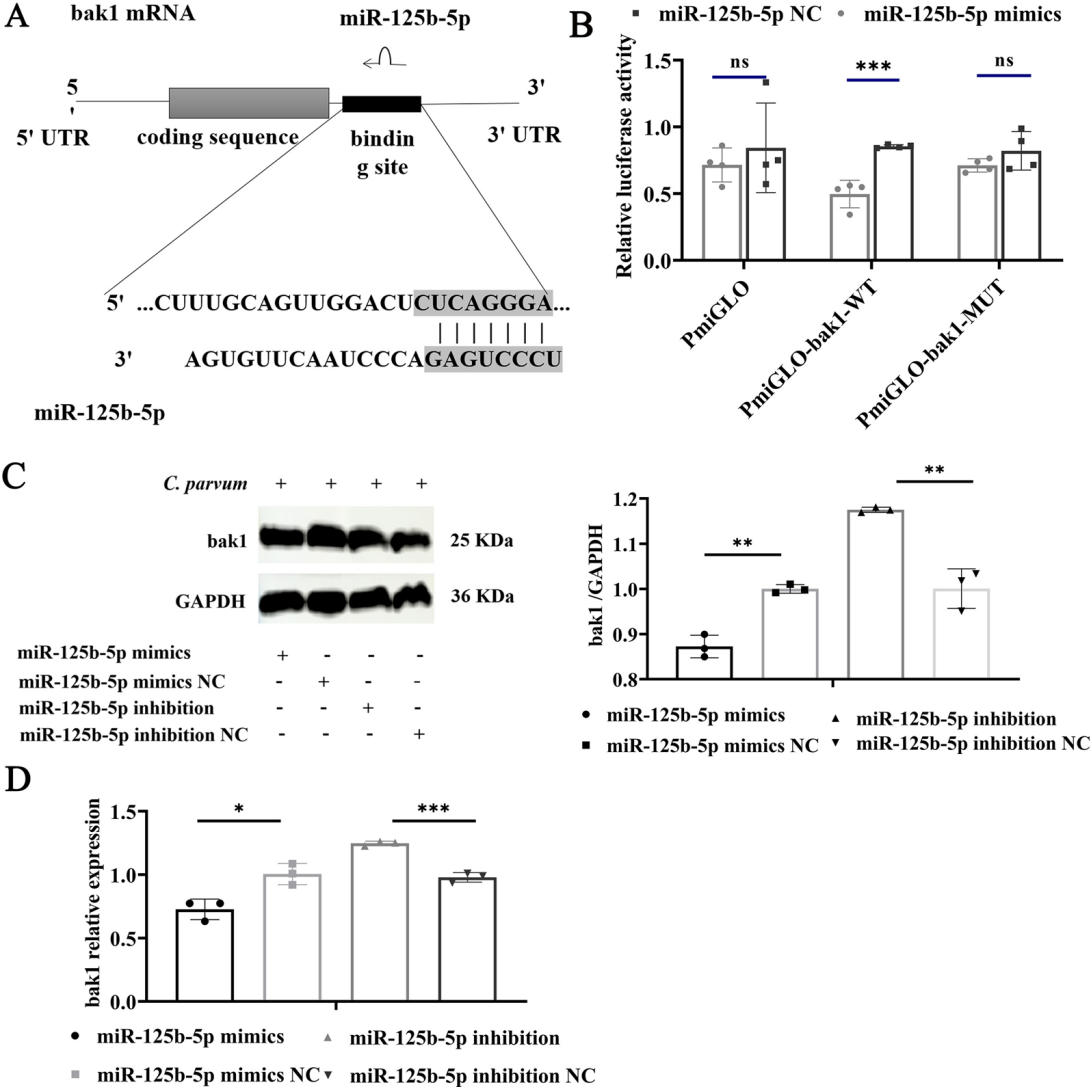
Bak1 is a target gene of miR-125b-5p. **A** Binding sites of miR-125b-5p on Bak1 predicted with TargetScan. **B** Binding of miR-125b-5p to Bak1 was detected with a dual-luciferase assay in HCT-8 cells cotransfected with miR-125b-5p or miR-125b-5p NC and pmirGLO–Bak1-WT or pmirGLO–Bak1-MUT for 48 h. **C, D** Cells were transfected with miR-125b-5p mimic or miR-125b-5p inhibitor for 24 h and then exposed to equal numbers of *C*. *parvum* sporozoites for 12 h. Bak1 mRNA and protein expression levels were detected with RT–qPCR and western blotting, respectively. Densitometric levels of Bak1 protein signals and mRNA levels were quantified and expressed relative to GAPDH. All data presented are the means ± SD of three independent experiments. Different groups were compared with a *t* test (*P < 0.05, **P < 0.01, ***P < 0.001)

Utilizing the bioinformatics tool TargetScan, we identified a potential binding site for miR-125b-5p within the 3′ untranslated region (UTR) of the Bak1 gene (Fig. 2a). To validate this interaction, we conducted a luciferase reporter assay. Transfecting HCT-8 cells with a pmirGLO vector containing the wild-type (WT) Bak1 target sites, along with the miR-125b-5p mimic, resulted in a significant decrease in luciferase activity. In contrast, cells transfected with a mutated Bak1 (MUT) construct or the vector alone did not exhibit significant changes in luciferase activity when combined with miR-125b-5p (Fig. 2b). Additionally, increasing miR-125b-5p expression via mimics lowered both mRNA and protein levels of Bak1, while inhibiting miR-125b-5p expression led to an increase in Bak1 levels (Fig. 2c, d). These findings confirm that miR-125b-5p directly targets Bak1, suppressing its expression, and thereby influencing apoptosis regulation in HCT-8 cells in response to *C*. *parvum* infection.

### Role of Bak1 in *C*. *parvum*-induced apoptosis in HCT-8 cells

**Fig. 3:**
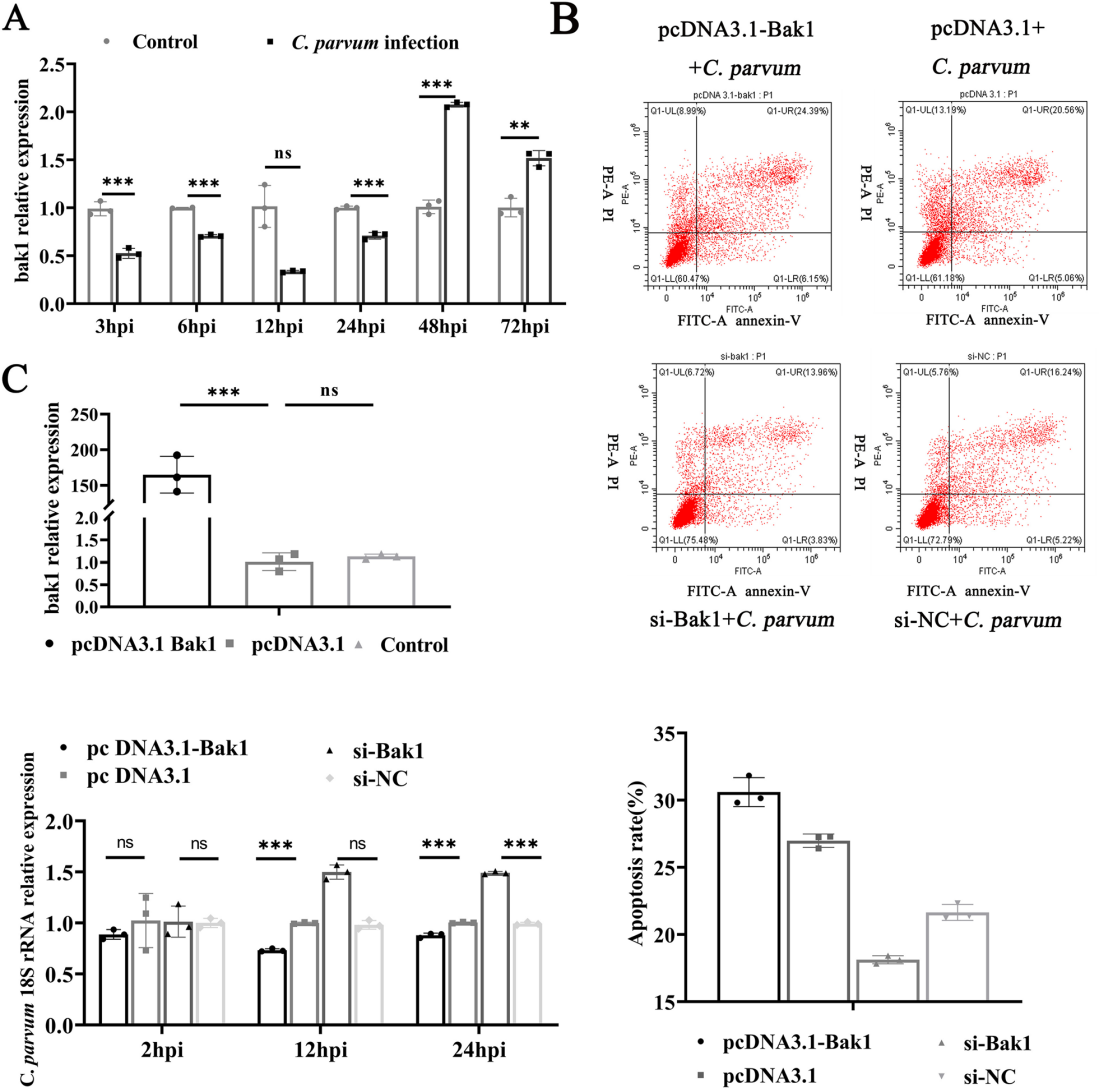
Bak1 is involved in the regulation of C. parvum-induced apoptosis in HCT-8 cells. **A** Expression levels of Bak1 in HCT-8 cells at different times after *C*. *parvum* sporozoites infection, determined with RT-qPCR. **B** Cells were transfected with pcDNA3.1-Bak1, pcDNA3.1 and si-Bak1 or si-NC for 24 h and then exposed to equal numbers of *C*. *parvum* sporozoites for 12 h. Cell apoptosis was then detected with flow cytometry. The horizontal axis of each apoptosis diagram is Annexin V and the vertical axis is PI; and the apoptosis rate is the sum of O1-LR and O1-UR. **C** Cells were transfected with pcDNA3.1-Bak1, pcDNA3.1 and si-Bak1 or si-NC for 24 h and then exposed to equal numbers of *C*. *parvum* sporozoites for 2 h before the medium was replaced with fresh medium. The infected cells were cultured for 12 or 24 h to evaluate the parasite burden with RT–qPCR after cell invasion. All data presented are the means ± SD of three independent experiments. Different groups were compared with a *t* test (*P < 0.05, **P < 0.01, ***P < 0.001)

RT-qPCR analysis revealed that Bak1 mRNA expression was significantly downregulated at 3, 6, 12 and 24 hpi; and notably upregulated at 48 hpi, following *C*. *parvum* infection (Fig. 3a). These observations suggest that *C*. *parvum* infection results in temporally differential expression of Bak1 in host cells. To explore Bak1’s role in apoptosis and parasite burden regulation during *C*. *parvum* infection, we designed three small interfering RNAs (siRNAs) targeting Bak1 and identified the most efficient siRNA (si-Bak1) for Bak1 silencing (Additional file 2: Fig. S1). Flow cytometry analysis showed a significant increase in apoptosis rates in HCT-8 cells transfected with pcDNA3.1-Bak1 compared with negative control-transfected cells, whereas si-Bak1 transfection led to decreased apoptosis compared with si-NC groups (Fig. 3b). Furthermore, RT-qPCR was utilized to assess Bak1’s functional role in modulating the *C*. *parvum* burden. At 2 hpi, Bak1 expression did not affect parasite load, but at 24 hpi, the si-Bak1 group showed a significant increased in parasite load, whereas the pcDNA3.1-Bak1 group demonstrated a significant decrease in parasite load (Fig. 3c). These results indicate that Bak1 plays a crucial role in regulating *C*. *parvum*-induced apoptosis in HCT-8 cells.

### miR-125b-5p regulates cell apoptosis and parasite burden in HCT-8 cells by targeting Bak1

**Fig. 4:**
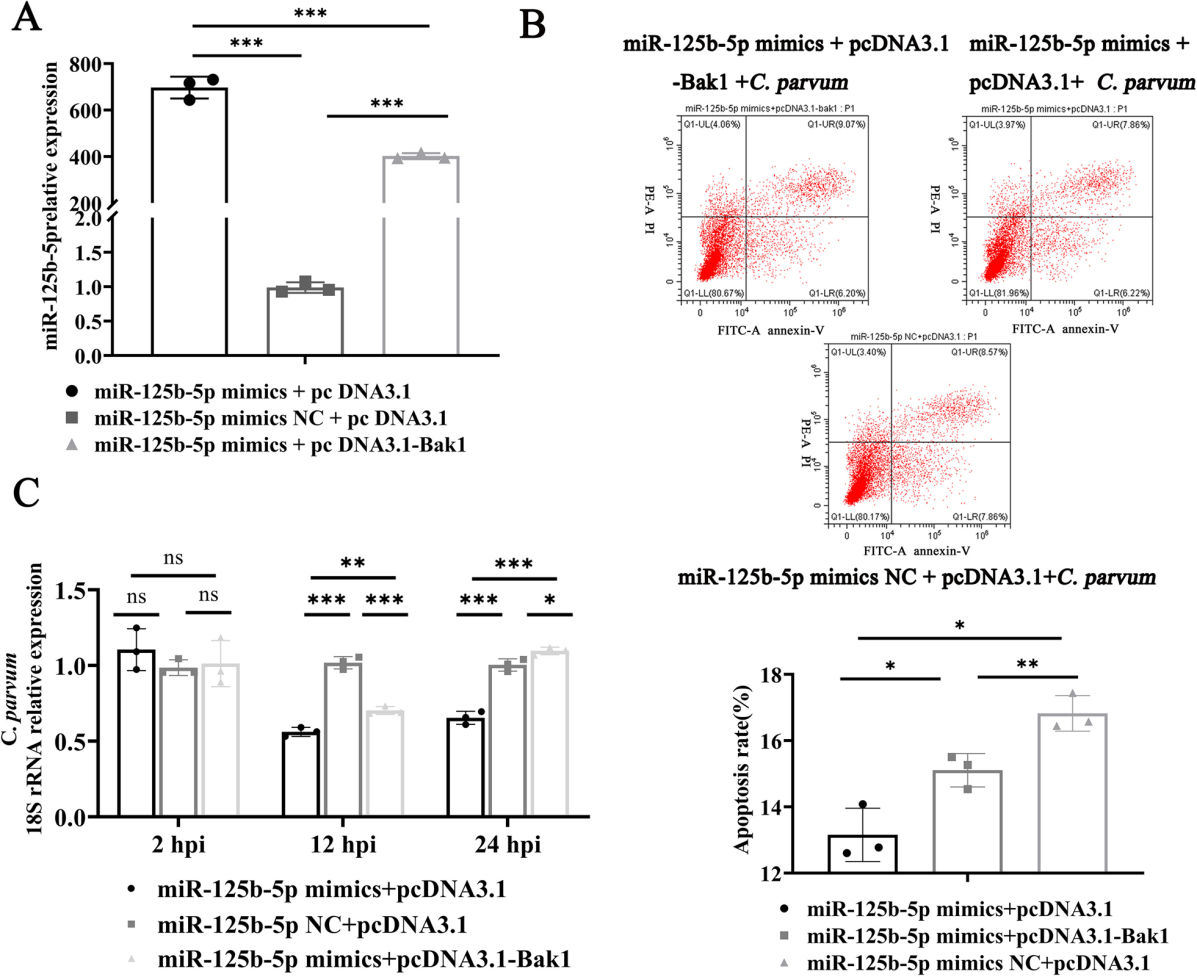
MiR-125b-5p regulates cell apoptosis and parasite burden in HCT-8 cells by targeting Bak1. **A** Expression efficiency of miR-125b-5p in HCT-8 cells cotransfected with miR-125b-5p mimics and pcDNA3.1-Bak1 for 24 h, determined with RT–qPCR. **B** Cells were cotransfected with miR-125b-5p mimics or miR-125b-5p mimics NC and pcDNA3.1-Bak1 or pcDNA3.1 for 24 h, and then exposed to equal numbers of *C*. *parvum* sporozoites for 12 h. Cell apoptosis was then detected with flow cytometry. The horizontal axis of each apoptosis diagram is Annexin V and the vertical axis is PI; and the apoptosis rate is the sum of O1-LR and O1-UR. **C** Cells were cotransfected with miR-125b-5p mimics or miR-125b-5p mimics NC and pcDNA3.1-Bak1 or pcDNA3.1 for 24 h were then exposed to equal numbers of *C*. *parvum* sporozoites for 2 h. The medium was then replaced with fresh medium. The infected cells were cultured for 12 or 24 h to evaluate the parasite burden after cell invasion, with RT-qPCR. All data presented are the means ± SD of three independent experiments. Different groups were compared with a *t* test (*P < 0.05, **P < 0.01, ***P < 0.001)

Previous findings demonstrated that miR-125b-5p mimics inhibit Bak1 expression, reducing apoptosis rates and increasing the *C*. *parvum* burden. Conversely, overexpression of Bak1 via pcDNA3.1-Bak1 increases apoptosis rates and decreases the *C*. *parvum* burden. To assess whether pcDNA3.1-Bak1 could counteract the effects of miR-125b-5p mimics and to confirm miR-125b-5p’s role in targeting Bak1, we co-transfected HCT-8 cells with miR-125b-5p mimics and pcDNA3.1-Bak1. Transfection efficiency was confirmed (Fig. 4a). Cells were then infected with *C*. *parvum* sporozoites to evaluate apoptosis and parasite burden. Flow cytometry analysis revealed that the apoptosis rate was significantly lower in cells co-transfected with miR-125b-5p mimics and pcDNA3.1 as opposed to those transfected with the negative controls, indicating miR-125b-5p’s role in reducing apoptosis. However, co-transfection with miR-125b-5p mimics and pcDNA3.1-Bak1 ameliorated this inhibition, illustrating that pcDNA3.1-Bak1 can reverse the regulatory effects of miR-125b-5p mimics (Fig. 4b). RT-qPCR analysis showed no changes in parasite burden across groups at 2 hpi. At 12 and 24 hpi, an increased parasite burden was observed in the miR-125b-5p mimics and pcDNA3.1 groups compared with their respective controls. Nonetheless, the increase was less pronounced in the co-transfected miR-125b-5p mimics and pcDNA3.1-Bak1 group, indicating that pcDNA3.1-Bak1 can mitigate the increase in parasite burden induced by miR-125b-5p mimics (Fig. 4c). These results confirm that miR-125b-5p targets Bak1 to limit apoptosis, thereby enhancing the *C*. *parvum* burden in HCT-8 cells.

### miR-125b-5p participates in regulating cell apoptosis via the mitochondrial pathway following *C*. *parvum* infection

**Fig. 5:**
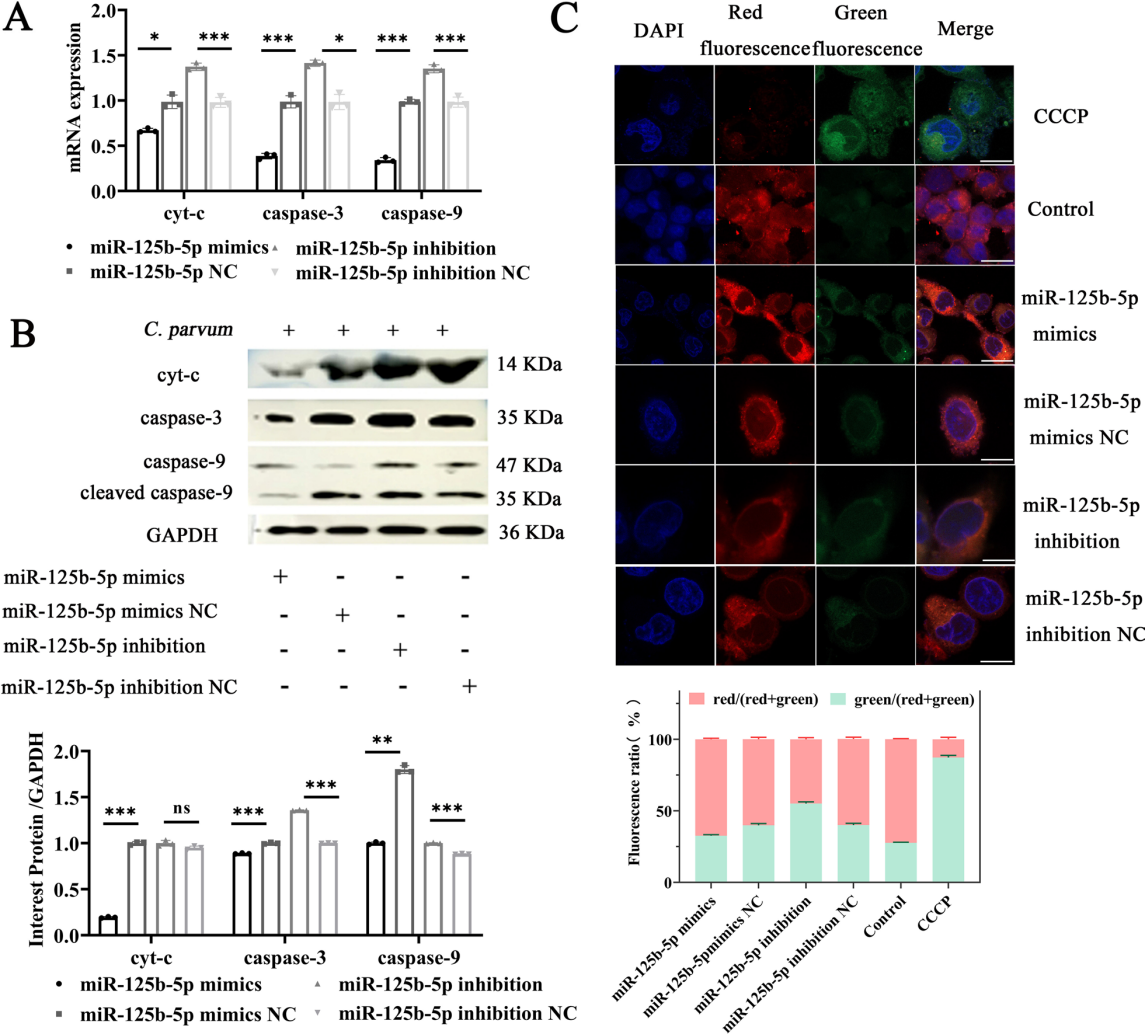
MiR-125b-5p participate in regulation cell apoptosis via mitochondrial pathway after *C*. *parvum* infection. **A, B** Cells were transfected with miR-125b-5p mimic or miR-125b-5p inhibitor for 24 h and then exposed to equal numbers of *C*. *parvum* sporozoites for 12 h. RT-qPCR and western blotting was used to detect the changes in mitochondrial pathway associated-molecules, Cytochrome-c (cyt-c), caspase-3 and caspase-9 at 12 hpi. **C** Cells were transfected with miR-125b-5p mimic or miR-125b-5p inhibitor for 24 h and then exposed to equal numbers of *C*. *parvum* sporozoites for 12 h. The changes of mitochondrial membrane potential were also evaluated by JC-1 probe and fluorescence microscopy assay and relative ratio of red fluorescence to green fluorescence can reflect the relative level of mitochondrial membrane potential. The enhanced red fluorescence indicated higher mitochondrial membrane potential and normal cell status; the enhancement of green fluorescence indicated that mitochondrial membrane potential decreased and cell apoptosis occurred (Scale bar, 10 µm). All data presented are the means ± SD of three independent experiments. Different groups were compared with a*t* test (*P < 0.05, **P < 0.01, ***P < 0.001)

To investigate whether miR-125b-5p regulates apoptosis through the mitochondrial pathway, we used miR-125b-5p mimics and inhibitors to modulate its expression. RT-qPCR and Western blotting were performed to detect changes in mitochondrial pathway-associated molecules—Cytochrome c (Cyt c), Caspase-3, and Caspase-9 at 12 hpi—and mitochondrial membrane potential changes were assessed using the JC-1 probe assay. Results indicated that overexpression of miR-125b-5p significantly decreased the mRNA and protein levels of Cyt c, Caspase-3, and Caspase-9 compared with controls, while inhibition of miR-125b-5p led to increased levels of these molecules (Fig. 5a, b). JC-1 probe assay results demonstrated that miR-125b-5p overexpression significantly increased the red fluorescence ratio compared with control, while its inhibition had the opposite effect, suggesting miR-125b-5p’s role in mitigating mitochondrial membrane damage (Fig. 5c). These findings indicate that miR-125b-5p regulates apoptosis via the mitochondrial pathway in *C*. *parvum*-infected HCT-8 cells.

### miR-125b-5p targets Bak1 to regulate apoptosis via the mitochondrial pathway in response to *C*. *parvum* infection

**Fig. 6:**
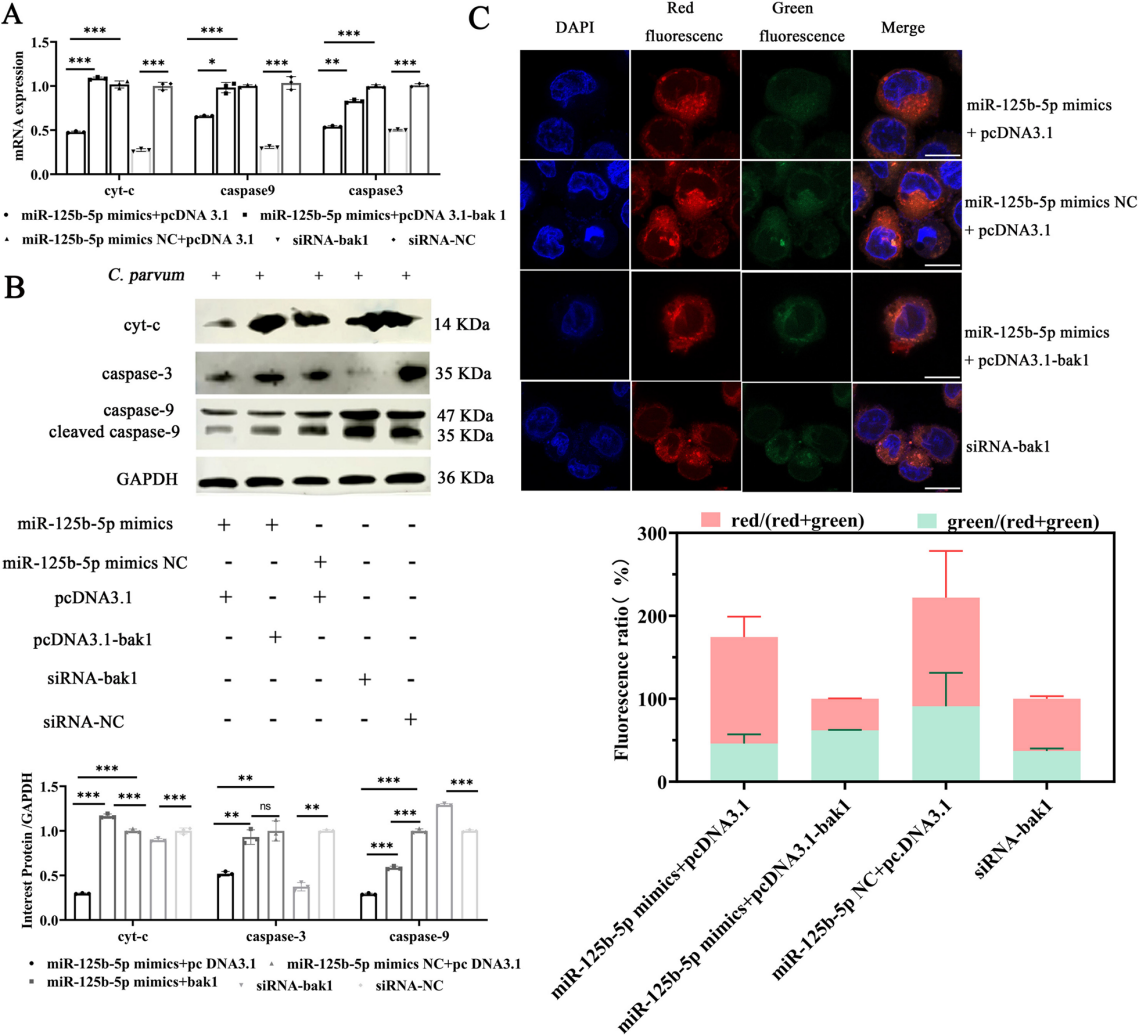
MiR-125b-5p targets Bak1 to regulate cell apoptosis in response to *C. parvum* infection via mitochondrial pathway. **A, B** Cells were were transfected si-Bak1 or si- NC or cotransfected were transfected with with miR-125b-5p mimics or miR-125b-5p mimics NC and pcDNA3.1-Bak1 or pcDNA3.1-Bak1 NC for 24 h, and then exposed to equal numbers of *C*. *parvum* sporozoites for 12 h. RT-qPCR and western blotting was used to detect the changes in mitochondrial pathway associated-molecules, Cytochrome-c (cyt-c), caspase-3 and caspase-9 at 12 hpi. **C** Cells were were transfected si-Bak1 or si- NC or cotransfected were transfected with with miR-125b-5p mimics or miR-125b-5p mimics NC and pcDNA3.1-Bak1 or pcDNA3.1-Bak1 NC for 24 h, and then exposed to equal numbers of *C*. *parvum* sporozoites for 12 h. The changes of mitochondrial membrane potential were also evaluated by JC-1 probe and fluorescence microscopy assay and relative ratio of red fluorescence to green fluorescence can reflect the relative level of mitochondrial membrane potential. The enhanced red fluorescence indicated higher mitochondrial membrane potential and normal cell status; the enhancement of green fluorescence indicated that mitochondrial membrane potential decreased and cell apoptosis occurred (Scale bar, 10 µm). All data presented are the means ± SD of three independent experiments. Different groups were compared with a *t* test (*P < 0.05, **P < 0.01, ***P < 0.001)

To confirm whether miR-125b-5p targets Bak1 to regulate apoptosis through the mitochondrial pathway, HCT-8 cells were co-transfected with miR-125b-5p mimics and pcDNA3.1-Bak1, then infected with *C*. *parvum* sporozoites for 12 h. We assessed the expression of Cytochrome c (Cyt c), Caspase-3, and Caspase-9, alongside evaluating mitochondrial membrane potential changes. RT-qPCR and Western blot analyses revealed that the mRNA and protein levels of Cyt c, Caspase-3, and Caspase-9 were significantly decreased in cells co-transfected with miR-125b-5p mimics and pcDNA3.1 compared with the miR-125b-5p mimic NC and pcDNA3.1 control group. However, these inhibitory effects were mitigated when cells were co-transfected with both miR-125b-5p mimics and pcDNA3.1-Bak1, indicating that pcDNA3.1-Bak1 can reverse miR-125b-5p’s regulatory effects (Fig. 6a, b). The JC-1 probe assay showed a significant increase in the proportion of red fluorescence in the miR-125b-5p mimic and pcDNA3.1 groups compared with their respective controls, reflecting reduced mitochondrial membrane potential damage. However, the increase in red fluorescence was less pronounced in the co-transfected miR-125b-5p mimics and pcDNA3.1-Bak1 groups compared with the miR-125b-5p mimic and pcDNA3.1 groups, demonstrating that pcDNA3.1-Bak1 counteracted the effects of miR-125b-5p mimics on mitochondrial membrane stability (Fig. 6c).These findings support the conclusion that miR-125b-5p targets Bak1 to regulate apoptosis in HCT-8 cells following *C*. *parvum* infection, facilitating this process through the mitochondrial pathway.

## Discussion

*Cryptosporidium parvum* infection induces significant changes in the expression of various microRNAs (miRNAs) in host cells, which can in turn affect the *C*. *parvum* infection burden by modulating target gene expression [15, 16]. Previous study found that, miR-27b targeted inhibition of KSPP expression and thus decreased *C*. *parvum* infection burden [25]; miR-199a-3p inhibited the *C*. *parvum* burden in HCT-8 cells by targeting MTOR [24]. Conversely, miR-4521 enhances *C*. *parvum* propagation by targeting foxm1. These studies suggest that miRNAs can influence infection loads through their interactions with downstream genes [26]. In this study, we demonstrate that *C*. *parvum* infection upregulates miR-125b-5p expression. This microRNA targets Bak1, consequently suppressing apoptosis and increasing parasitic burden in HCT-8 cells. Previous studies indicate a biphasic modulation of apoptosis during *C*. *parvum* infection: initial suppression facilitates parasite establishment, while subsequent induction limits pathogen proliferation [6, 9]. Our findings reveal that miR-125b-5p is downregulated during the early phase (3–24 hpi), facilitating pro-apoptotic Bak1 expression, but significantly upregulated at later stages (48–72 hpi), inhibiting apoptosis. This temporal expression pattern aligns with the established biphasic model and suggests miR-125b-5p plays a key role in mediating the host’s dynamic apoptotic response. This mechanism may balance early parasite containment with the later mitigation of excessive cell death. The early downregulation of miR-125b-5p (3–24 hpi) likely represents an innate host defense strategy. By permitting Bak1-mediated apoptosis, the host potentially eliminates infected cells to curtail parasite replication. This is consistent with studies demonstrating that inhibiting apoptosis enhances *C*. *parvum* survival [9]. Conversely, the later induction of miR-125b-5p may reflect a parasite-driven subversion mechanism to prolong host cell viability, thereby promoting persistent infection. This intricate miR-125b-5p-mediated interaction represents a critical point in the host–parasite interplay during the immune response.

Apoptosis is a crucial defense mechanism, enabling host cells to execute innate immune functions against *C*. *parvum*[27]. Research indicates that miRNAs regulate host cell apoptosis in response to *C*. *parvum*[28, 29]. For instance, Yao et al. demonstrated that miR-4521 suppresses BCL-2-mediated apoptosis by targeting foxm1, promoting *C*. *parvum* propagation [26]. Meanwhile, our prior studies showed that miR-181d targets BCL2 to induce apoptosis, reducing the *C*. *parvum* burden [29], while miR-3976 enhances apoptosis by targeting BCL2A1, suppressing parasite loads [22]. Our current study reveals that miR-125b-5p inhibits apoptosis by targeting Bak1, thereby promoting *C*. *parvum* burden. These results establish miR-125b-5p as a novel regulator of mitochondrial apoptosis that specifically targets Bak1, thereby expanding the known regulatory network governing this pathway. The functional dichotomy of miRNAs—where pro-apoptotic factors (e.g., miR-181d) contrast with anti-apoptotic regulators (e.g., miR-125b-5p)—underscores their collective role in fine-tuning host–pathogen interactions. Notably, while both Bak1 and BCL2A1 belong to the BCL2 family, they have distinct roles: BCL2 and BCL2A1 primarily function as anti-apoptotic proteins, whereas Bak1, a pro-apoptotic BCL2 protein, induces apoptosis at the mitochondrial membrane [30–33]. MiRNAs can downregulate these genes post-*C*. *parvum* infection, suggesting that miRNAs impact HCT-8 cell apoptosis and, consequently, parasite proliferation through their target genes [22, 29, 34, 35]. These evidences suggest that microRNAs regulate the apoptosis of HCT-8 cells and thus affect parasite proliferation, which may be related to the apoptosis pathway mediated by the target genes they interact with.

Apoptosis can proceed via two primary pathways: the extrinsic pathway, dependent on death receptors, and the intrinsic/mitochondrial pathway mediated by the Bcl-2 family [5, 36]. Previous research demonstrated that *C*. *parvum* regulates death receptors (e.g., Fas/FasL, TRAI/TRAIL) to modulate host cell apoptosis, although the specific pathways regulated by miRNAs in response to *C*. *parvum* remain less explored [6, 37, 38]. Our previous findings highlighted that miR-199a-3p targets the MTOR signaling pathway, promoting autophagy and apoptosis in response to *C*. *parvum*[24]. MiR-942-5p influences the TLR2/4-NF-κB pathway to modulate IFI27 expression, impacting apoptosis via the TRAI/TRAIL pathway [23]. In addition, *C*. *parvum* infection altered the expression of miR-181d and BCL2 and activated the intrinsic apoptosis pathway in HCT-8 cells [29]. However, the exogenous pathway of apoptosis is mainly through the activation of promoter caspase-8, while the endogenous pathway is mainly dependent on the release of Cyt c and the activation of caspase-9 and caspase-3 in mitochondria [39]. In this study, we demonstrated that miR-125b-5p mimics reduce the expression of cytochrome c, caspase-3, and caspase-9, components critical in the mitochondrial apoptosis pathway. However, these effects were reversed when cells were also transfected with pcDNA3.1-Bak1, indicating that Bak1 can counteract miR-125b-5p’s inhibition. Bak1, a pro-apoptotic Bcl-2 family molecule, influences mitochondrial membrane permeability and cytochrome c release, a key step in initiating apoptosis [40]. JC-1 probe assays further showed that miR-125b-5p mimics reduced mitochondrial membrane damage, an effect reversed by pcDNA3.1-Bak1. These findings suggest that miR-125b-5p modulates apoptosis and *C*. *parvum* burden through Bak1 via the mitochondrial pathway. This study provides new insights into the complex interactions between host miRNAs and *Cryptosporidium*.

## Conclusion

In conclusion, the findings from this study suggest that *C*. *parvum* infection alters the expression of miR-125b-5p and Bak1 in HCT-8 cells. miR-125b-5p targets Bak1, affecting the parasite burden by regulating cell apoptosis through the mitochondrial pathway. While our study employed HCT-8 cells—a well-established in vitro model for cryptosporidiosis; however, the physiological context in vivo, including immune cell crosstalk and tissue-specific microenvironments, may influence the functional role of miR-125b-5p [14, 19]. Future studies using enteroid models or in vivo systems (e.g., murine or bovine infection models) are warranted to determine whether miR-125b-5p’s regulation of apoptosis is conserved across species. Future research should validate these mechanisms in vivo and explore potential therapeutic applications arising from these findings.

## Supplementary Information

**Additional file 1.**

**Additional file 2.**

## Data Availability

Data supporting the main conclusions of this study are included in the manuscript.

## Abbreviations

*C*. *parvum*: *Cryptosporidium parvum*
HCT-8 cells: Human ileocecal adenocarcinoma cells
3′-UTR: 3′-Untranslated region
KSRP: KH-type splicing regulatory protein
Fas: Factor-associated suicide
FasL: Fas ligand
RT-qPCR: Real-time quantitative polymerase chain reaction
Bak1: BCL2-antagonist/killer 1
Cyt c: Cytochrome Complex
Caspase-3: Cysteine protease protein 3
Caspase-9: Cysteine protease protein 9
Mimics: Synthetic miRNA analogs used to overexpression miRNA
Inhibitors: Antisense oligonucleotides used to knockdown miRNA
