# Supplementary material for: MiR-125b-5p targets Bak1 to regulate HCT-8 cell apoptosis in response to Cryptosporidium parvum infection via mitochondrial pathway
Source: Parasit Vectors. 2025 Oct 28;18:435. doi: 10.1186/s13071-025-07033-1 (PMC12570444; doi:10.1186/s13071-025-07033-1)

Supplementary information

Table S1. Primers used in RT-qPCR and sequences used to generate constructs

Target	Primers	
	**Forward**	**Reverse**
Human-SSU rRNA	CCGATAACGAACGAGACTCTGG	TAGGGTAGGCACACGCTGAGCC
C. parvum-SSU rRNA	TAGAGATTGGAGGTTGTTCCT	CTCCACCAACTAAGAACGGCC
Bak1	TACACGTCTACCAGCATGGC	CCAACAGAACCACACCCAGA
GAPDH	GTC AGC CGC ATC TTC TTT TG	GCG CCC AAT ACG ACC AAA TC
U6	TGG AAC GCT TCA CGA ATT TGC G	GGA ACG ATA CAG AGA AGA TTA GC
miR-125b-5p	TCCCTGAGACCCTAACTTGTGA	AGTCTCAGGGTCCGAGGTATTC
miR-125b-5p RT	GTCGTATCCAGTGCAGGGTCCGAGGTATTCGCACTGGATACGACTCACAA	

Table S2. RNA oligonucleotides for miRNAs and siRNAs

Target	Sense	Antisense
miR-125b-5p mimics	ACAGUAGUCUGCACAUUGGUUA	ACCAAUGUGCAGACUACUGUUU
miR-125b-5p mimics NC	UUC UCC GAA CGU GUC ACG UTT	ACG UGA CAC GUU CGG AGA
miR-125b-5p inhibitor	UAACCAAUGUGCAGACUACUGU	
miR-125b-5p inhibitor NC	CAG UAC UUU UGU GUA GUA CAA	
genOFFTM st-h-BAK1_001	GTTTGAGAGTGGCATCAAT	
genOFFTM st-h-BAK1_002	GGTGGTACGAAGATTCTTCA	
genOFFTM st-h-BAK1_003	GCTTCGTGGTCGACTTCAT	

Table S3. The inserted sequence of mTOR -WT and mTOR MUT

**Target**	**sense**
Bak1-WT	CTTTGCAGTTGGACTCTCAGGGACTTTGCAGTT<br>GGACTCTCAGGGACTTTGCAGTTGGACT
Bak1 -MUT	CTTTGCAGTTGGACTTGGCTCAGCTTTGCAGTTG<br>GACTTGGCTCAGCTTTGCAGTTGGACT

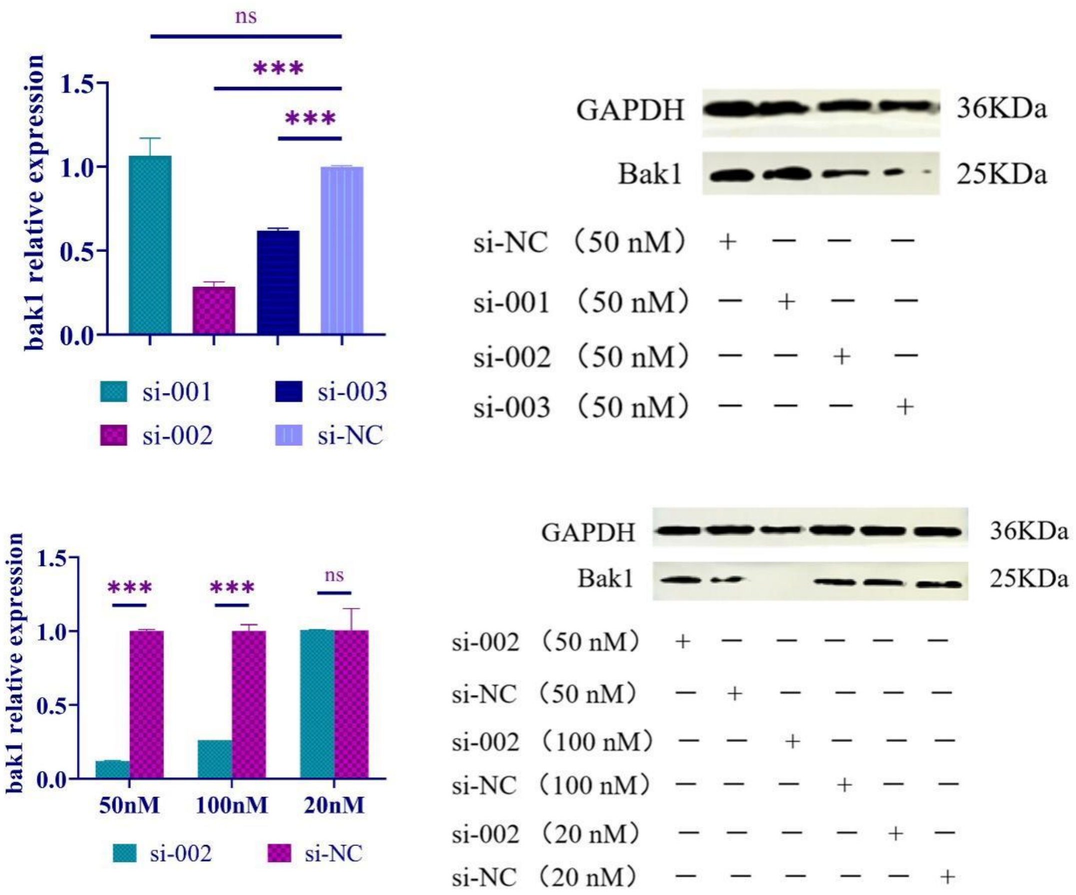

Supplementary Fig. 1 Optimal concentrations of three pairs of small interfering RNA (siRNA; si-Bak 1) were determined with RT–qPCR and Western blotting.

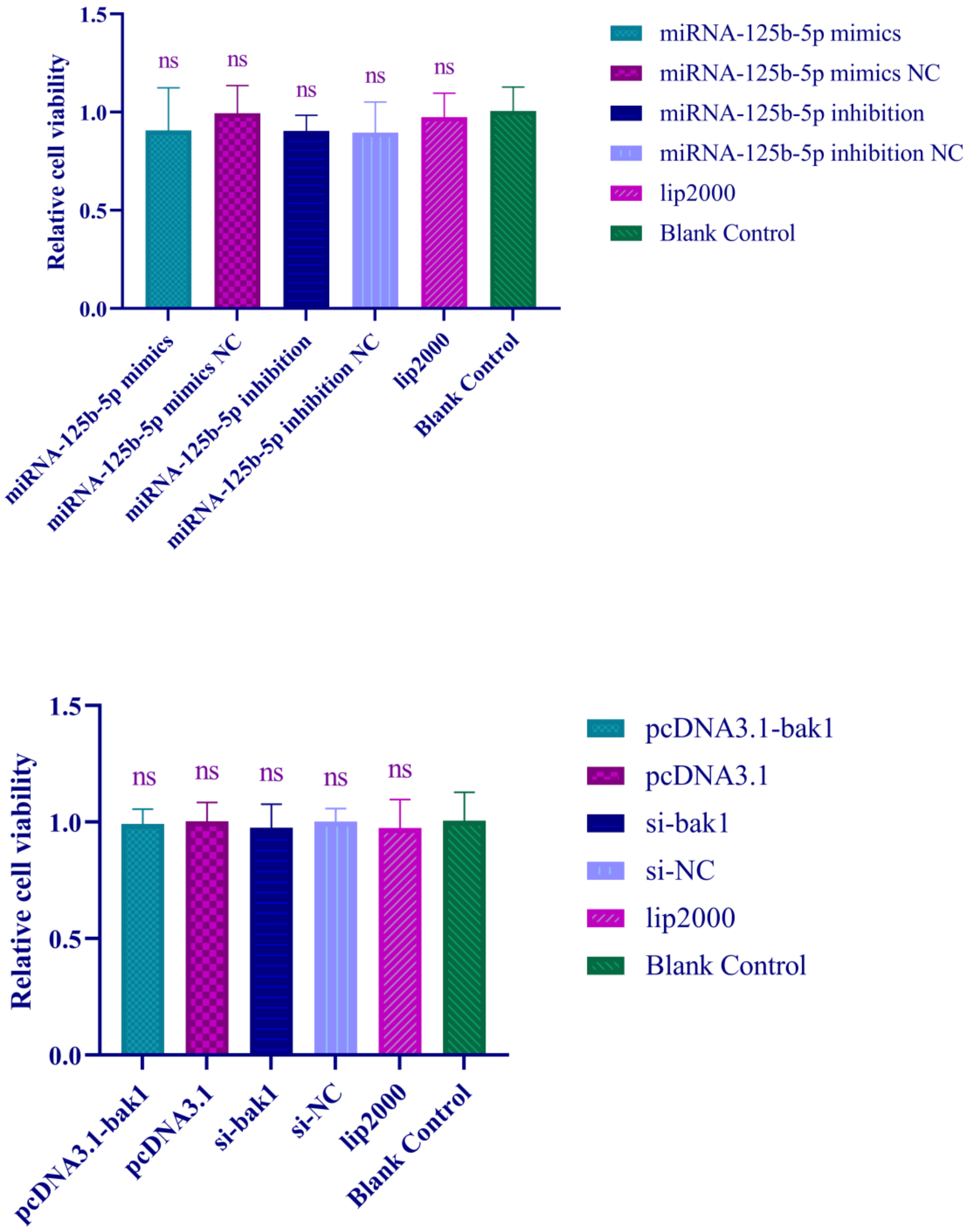

Supplementary Fig. 2 Effects of transfection of miR-125b-5p mimic and miR-125b-5p inhibitor and si-bak 1 or si-NC with Lip2000 on cell activity

Supplementary Fig. 3 Structure diagram of double luciferase carrier.

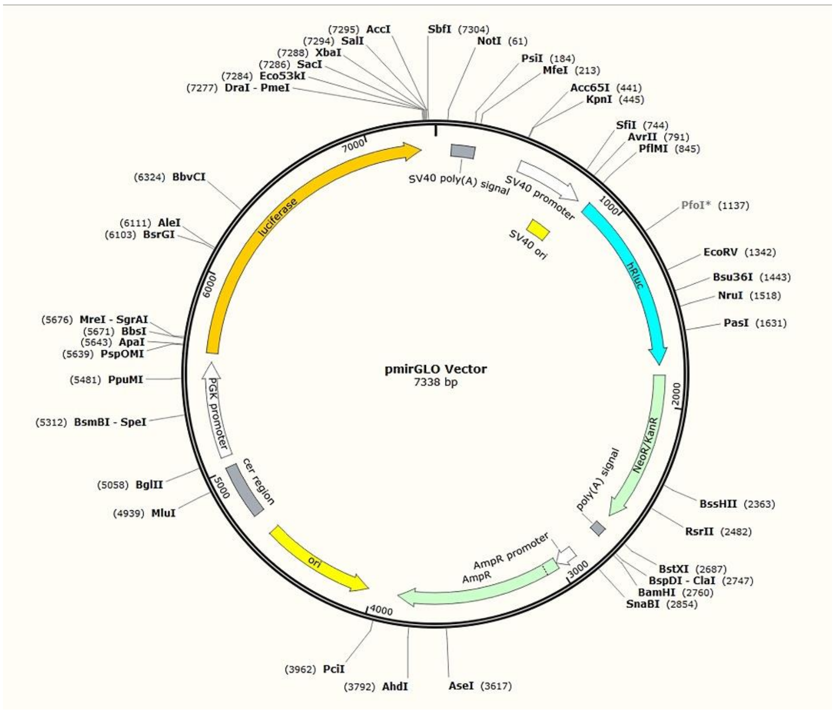

Supplementary Fig.4 Gating strategy for flow cytometry analysis.

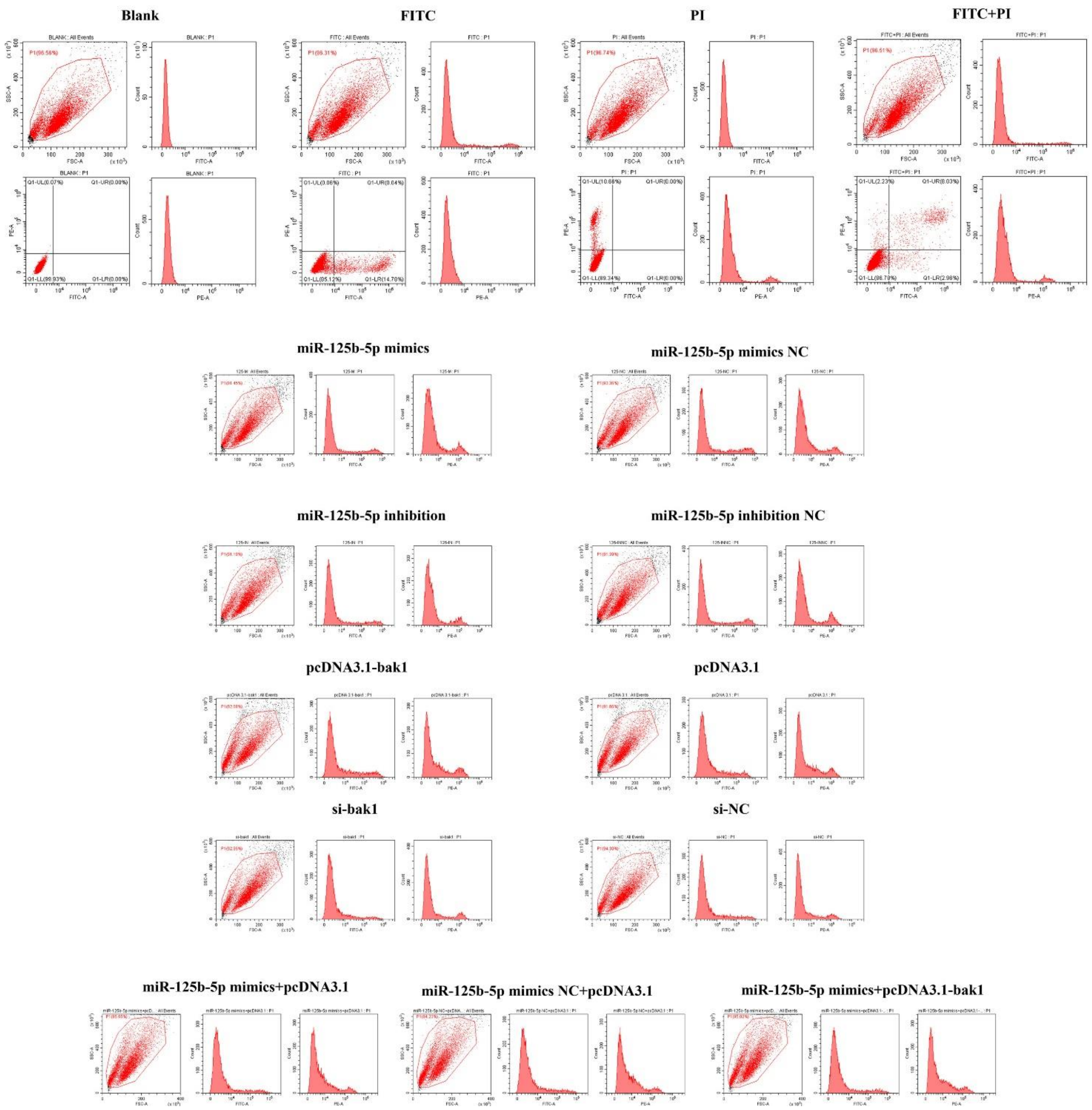

Supplementary Fig. 5 Uncropped blots images for western blot.

FIGURE 2C

bak1

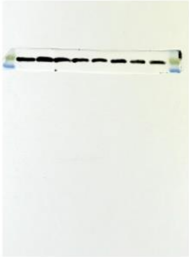

GAPDH

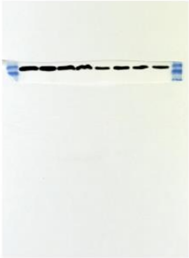

FIGURE 5B

cyt-c

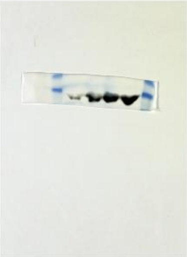

GAPDH

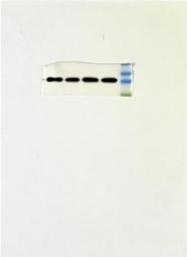

caspase-3

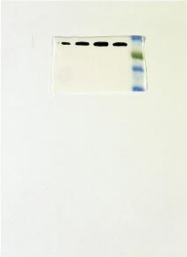

caspase-9

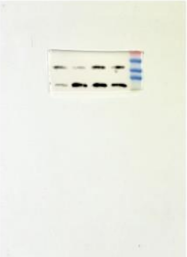

FIGURE 6B

GAPDH

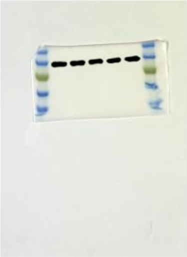

cyt-c

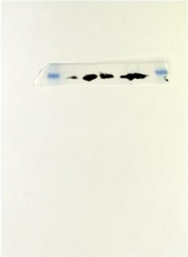

caspase-3

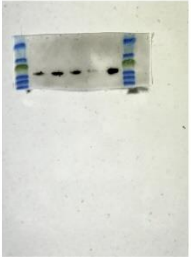

caspase-9

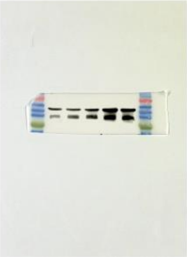

Supplement: Supplementary file 2 — Additional file 2. [file 13071_2025_7033_MOESM2_ESM.pdf]
